# Genetic Polymorphisms of Dihydropyrimidinase in a Japanese Patient with Capecitabine-Induced Toxicity

**DOI:** 10.1371/journal.pone.0124818

**Published:** 2015-04-27

**Authors:** Masahiro Hiratsuka, Hiroshi Yamashita, Fumika Akai, Hiroki Hosono, Eiji Hishinuma, Noriyasu Hirasawa, Takahiro Mori

**Affiliations:** 1 Laboratory of Pharmacotherapy of Life-Style Related Diseases, Graduate School of Pharmaceutical Sciences, Tohoku University, Sendai, Japan; 2 Department of Surgery, Iwate Prefectural Chubu Hospital, Kitakami, Japan; 3 Graduate School of Medicine, Tohoku University, Sendai, Japan; University de Minho, PORTUGAL

## Abstract

Dihydropyrimidinase (DHP) is the second enzyme in the catabolic pathway of uracil, thymine, and chemotherapeutic fluoropyrimidine agents such as 5-fluorouracil (5-FU). Thus, DHP deficiency might be associated with 5-FU toxicity during fluoropyrimidine chemotherapy. We performed genetic analyses of the family of a patient with advanced colon cancer who underwent radical colectomy followed by treatment with 5-FU prodrug capecitabine and developed severe toxicity attributable to a lack of DHP. We measured urinary uracil and dihydrouracil, and genotyped *DPYS* in the patient and her family. We also measured the allele frequency of *DPYS* polymorphisms in 391 unrelated Japanese subjects. The patient had compound heterozygous missense and nonsense polymorphisms comprising c.1001A>G (p.Gln334Arg) in exon 6 and c.1393C>T (p.Arg465Ter) in exon 8, which are known to result in a DHP enzyme with little or no activity. The urinary dihydrouracil/uracil ratio in the patient was 17.08, while the mean ± SD urinary dihydrouracil/uracil ratio in family members who were heterozygous or homozygous for wild-type *DPYS* was 0.25 ± 0.06. In unrelated subjects, 8 of 391 individuals were heterozygous for the c.1001A>G mutation, while the c.1393C>T mutation was not identified. This is the first report of a DHP-deficient patient with *DPYS* compound heterozygous polymorphisms who was treated with a fluoropyrimidine, and our findings suggest that polymorphisms in the *DPYS* gene are pharmacogenomic markers associated with severe 5-FU toxicity in Japanese patients.

## Introduction

**Fig 1:**
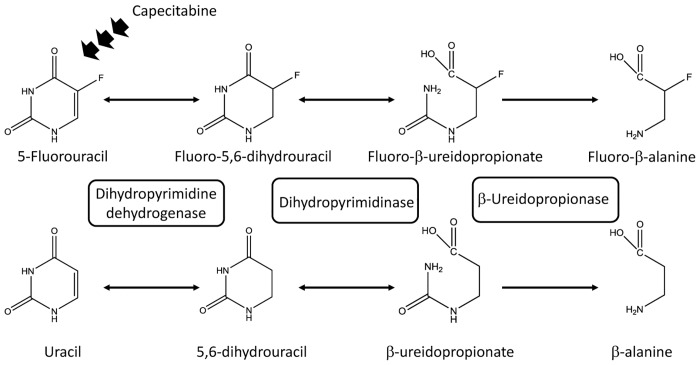
Catabolic pathway of 5-fluorouracil and uracil. 5-FU and uracil are inactivated by dihydropyrimidine dehydrogenase to fluoro-5,6-dihydrouracil and 5,6-dihydrouracil, respectively, which are subsequently converted by dihydropyrimidinase to fluoro-β-ureidopropionate and β-ureidopropionate, respectively. Fluoro-β-alanine and β-alanine are the final catabolite in this cascade and are formed by β-ureidopropionase.

5-Fluorouracil (5-FU) and its oral prodrug capecitabine are fluoropyrimidines, which are among the most commonly prescribed anticancer drugs for various types of solid tumors [1]. 5-FU is metabolically unstable in humans, so capecitabine is used as a prodrug that is converted to 5-FU by several enzymes within tumor tissues [1]. The majority of 5-FU is inactivated by dihydropyrimidine dehydrogenase (DPD), mainly in the liver, to dihydro-5,6-fluorouracil. Subsequently, dihydropyrimidinase (DHP) catalyzes the hydrolytic ring opening of dihydro-5,6-fluorouracil. Finally, the resulting fluoro-β-ureidopropionate is converted to fluoro-β-alanine by β-ureidopropionase [2]. The enzymes of the 5-FU degradation pathway are also involved in the degradation of uracil (Fig 1).

5-FU toxicity is largely dependent on its catabolism [1]. It has been reported that DPD-deficient patients are at an increased risk of developing severe toxicity after 5-FU administration [3–6]. However, there are few reports of patients with a DHP deficiency who developed severe 5-FU-associated toxicity [7–9]. DHP is encoded by the *DPYS* gene on chromosome 8q22, which consists of 10 exons containing a 1560-bp open reading frame and encodes a polypeptide of 519 amino acid residues [10]. DHP deficiency is an autosomal recessive disease characterized by an accumulation of dihydrouracil and dihydrothymine in the urine, blood, and cerebrospinal fluid [7, 10–18]. Although symptomatic patients show dysmorphic features and experience seizures, mental retardation, and growth retardation, asymptomatic patients have been identified, and the clinical, biochemical, and genetic spectrum of DHP-deficient patients remains unclear. DHP-deficient individuals might remain asymptomatic until treated with 5-FU or a prodrug.

Here, we report a patient with severe capecitabine-associated toxicity and DHP deficiency caused by compound heterozygous missense and nonsense polymorphisms in the *DPYS* gene, as well as genetic analysis of the family of this patient. Furthermore, we searched for genetic variations in *DPYS* by sequencing its exons from 391 unrelated Japanese subjects.

## Materials and Methods

### Subjects

A 53-year-old Japanese woman underwent ileocecal resection for cancer in the cecum, which was diagnosed as T4aN1bM0 stage-IIIB according to the TNM classification (7th edition; UICC) [19]. The patient was treated with capecitabine as postoperative adjuvant chemotherapy, because postoperative administration of capecitabine has been proven to reduce the risk of colon cancer recurrence [20]. Capecitabine (1,250 mg/m^2^) was orally administered twice daily as recommended in the treatment guidelines (JSCCR Guidelines 2014 for the Treatment of Colorectal Cancer in Japan, National Comprehensive Cancer Network (NCCN) guidelines version 2.2015 colon cancer, principles of adjuvant therapy (http://www.nccn.org/professionals/physician_gls/pdf/colon.pdf)). The patient visited the hospital after developing oral mucositis, and the oncologist stopped capecitabine treatment 10 days after initiation. Nevertheless, the patient experienced vomiting, severe diarrhea, and high fever, and she was hospitalized and treated with antibiotics and a granulocyte-colony stimulating factor for these conditions. However, her general condition rapidly worsened to disseminated intravascular coagulation and loss of consciousness. The patient underwent hemodialysis to remove toxic compounds for 2 days, her condition subsequently improved, and she was eventually discharged from the hospital. A case report describing the patient’s detailed clinical course is in preparation, including surgeons’ operative findings and laboratory data.

### Analysis of pyrimidines in urine

Urine samples from the patient and other subjects were collected after the patient recovered (25 days after initiation of capecitabine treatment) and during working hours (9 A.M.–5 P.M.), respectively. The concentrations of uracil and dihydrouracil in urine were analyzed by FALCO biosystems (Kyoto, Japan). Urine was filtered using a centrifuge for 5 min at 10000 r.p.m. (7200 × *g*) with a centrifugal filter and subjected to high-performance liquid chromatography with a column switching system. The first column was a Develosil ODS-5 column (length, 150 mm; inside diameter, 6.0 mm; Nomura chemical, Tokyo, Japan) and the second column was an MCI GEL column (length, 300 mm; inside diameter, 8.0 mm; Mitsubishi Chemical, Tokyo, Japan). The elution from the second column was monitored at 210 nm and 260 nm for uracil and dihydrouracil, respectively.

### Polymerase chain reaction (PCR) amplification of coding exons and sequence analysis

**Fig 2:**
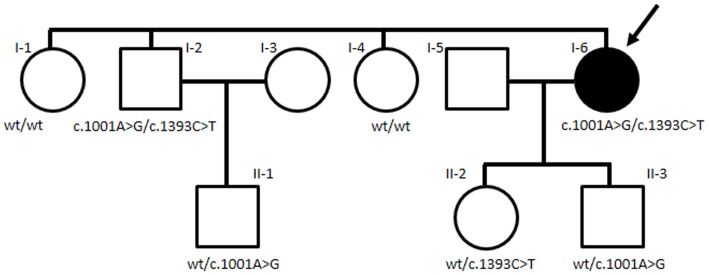
Pedigree of the patient with severe 5-FU toxicity. The arrow indicates the patient with severe toxicity after capecitabine treatment.

To identify the family genotype and confirm sequence alterations, we performed DNA analysis by PCR amplification using peripheral blood leukocyte genomic DNA from the proband (I-6), as well as other available members of her family (her sisters I-1 and I-4, her brother I-2, her nephew II-1, her daughter II-2, and her son II-3) (Fig 2).

For population screening, we recruited 391 unrelated subjects at Tohoku University Hospital from December 2008 to December 2010. All participants including healthy volunteers and hospital patients who had urological diseases were members of the Japanese population aged between 33 and 94 years (52 females and 339 males). Genomic DNA was prepared from whole blood using QIAamp DNA Blood Mini Kits (Qiagen, Hilden, Germany) in accordance with the manufacturer’s instructions. The local Ethics Committee of Tohoku University School of Medicine and Tohoku University Hospital approved the study, and all blood donors provided written informed consent. The primer pairs used to amplify exons 1–23 of the *DPYD* gene and exons 1–9 of the *DPYS* gene are listed in S1 and S2 Tables. The primers were designed on the basis of genomic sequences reported in GenBank (NG_008807.2 for *DPYD* and NG_008840.1 for *DPYS*). The reaction mixture contained approximately 30 ng genomic DNA, 0.5 μM of each primer, and 10× AmpliTaq Gold PCR Master Mix (Applied Biosystems, Foster City, CA, USA) in a total reaction volume of 20 μL. PCR for *DPYD* exons 1, 2, 4, 5, and 7–22 was performed as reported previously [21, 22]: initial denaturation at 95°C for 10 min was followed by 12 cycles of denaturation at 92°C for 30 s, annealing at decreasing temperatures stepwise from 60°C to 55°C for 30 s, extension at 70°C for 30 s, 18 additional cycles with an annealing temperature of 50°C, and a final extension of 7 min at 70°C. Because exons 3, 6, and 23 were not successfully amplified with this protocol, the annealing temperature was modified to 50°C for 30 repetitive cycles. The PCR conditions for *DPYS* exons comprised an initial denaturation at 95°C for 10 min, followed by 30 repetitive cycles of denaturation at 95°C for 30 s, annealing for 30 s, and extension at 72°C for 1 min, and a final extension at 72°C for 7 min. The annealing temperatures were as follows: 63°C for exon 1, 60°C for exons 2–6, 8, and 9, and 50°C for exon 7. The resulting amplification products were purified using the FastGene PCR Extraction Kit (NIPPON Genetics Co., Tokyo, Japan). Dye-terminator cycle sequencing was performed using the BigDye Terminator v3.1 Cycle Sequencing Kit (Applied Biosystems, Foster City, CA, USA). The amplicons were sequenced in both directions with an Applied Biosystems 3730xl DNA analyzer (Applied Biosystems).

## Results

### Analysis of pyrimidines in urine

To investigate whether severe toxicity in the patient (subject I-6 in Fig 2) treated with capecitabine might have been caused by a deficiency of DPD and/or DHP, urine was collected for measurement of the levels of uracil and dihydrouracil. As shown in Table 1, the urinary dihydrouracil concentration in subject I-6 (the proband) was 1708.0 μmol/g creatinine, which was more than 95-fold of reported normal control levels. Similar to subject I-6, subject I-2 (proband’s brother) showed a urinary dihydrouracil concentration higher (1664.9 μmol/g creatinine) than that of the other family members and reported normal control levels. The urinary dihydrouracil/uracil ratios in subject I-6 and subject I-2 were 17.08 and 7.91, respectively. In contrast, the urinary dihydrouracil/uracil ratio in other family members of the patient was 0.25 ± 0.06 (mean ± SD).

**Table 1:** Urinary pyrimidine concentrations and DPYS genotypes in the family of the patient with severe 5-FU toxicity.

			Urinary pyrimidine (μmol/g creatinine)			DPYS polymorphisms	
Subjects	Age	Sex	Uracil (U)	Dihydrouracil (DHU)	DHU/U ratio	Genotype	Effect
I-6	56	F	100.0	1708.0	17.08	c.1001A>G^c^ /c.1393C>T	p.Gln334Arg/p.Arg465Ter
II-2	31	F	64.9	18.0	0.28	wt^b^/c.1393C>T	wt/p.Arg465Ter
II-3	29	M	55.8	16.3	0.29	wt/c.1001A>G^c^	wt/p.Gln334Arg
I-1	67	F	66.5	12.8	0.19	wt/wt	wt/wt
I-2	63	M	210.6	1664.9	7.91	c.1001A>G^c^ /c.1393C>T	p.Gln334Arg/p.Arg465Ter
I-4	60	F	58.2	18.5	0.32	wt/wt	wt/wt
II-1	29	M	51.5	9.0	0.17	wt/c.1001A>G^c^	wt/p.Gln334Arg
Control^a^			66.3 ± 30.0	17.5 ± 5.3	0.26		

^a^ Kouwaki et al. ***Clinical Cancer Research***, 4: 2999–3004 (1998) [27]

^b^ wt, wild-type

^c^c.1001A>G was in cis with common synonymous variant c.216C>T (p.F72F).

### Sequence analysis of the DPYD and DPYS genes

With regard to the genomic DNA of subject I-6, sequence analysis of *DPYD* exons 1–23 and exon-intron boundaries showed no nonsynonymous substitutions or splice variants (including c.1905+1G>A), although the synonymous substitution c.1896T>C (rs17376848) (p.Phe632Phe) was detected. By sequence analysis of *DPYS* exons 1–9 and exon-intron boundaries in the proband’s DNA, the missense polymorphism c.1001A>G (rs121964923) in exon 6 and the nonsense polymorphism c.1393C>T (rs201280871) in exon 8 were found (S1 Fig). The c.1001A>G polymorphism results in the amino acid substitution p.Gln334Arg, whereas the c.1393C>T polymorphism results in the generation (p.Arg465Ter) of a stop codon (Table 1). As shown in Fig 2 and Table 1, family screening revealed that subject I-2 had the same *DPYS* genotype as the patient. Subjects I-1 and I-4 (proband’s sisters) did not possess the polymorphisms identified in exons 6 and 8. However, subject II-2 (proband’s daughter) was heterozygous for the c.1393C>T polymorphism, and subjects II-3 (proband’s son) and II-1 (proband’s nephew) were heterozygous for the c.1001A>G polymorphism. The proband’s children were heterozygous for the c.1393C>T or c.1001A>G polymorphisms, indicating that the proband and her brother (I-2) are likely compound heterozygotes of c.1001A>G and c.1393C>T.

### Population screening for the c.1001A>G and c.1393C>T polymorphisms

Screening of individuals for the presence of the c.1001A>G and c.1393C>T polymorphisms was performed using Sanger sequencing method. Eight of the 391 unrelated Japanese individuals were heterozygous for the c.1001A>G polymorphism, and therefore, the frequency of the allele in the study population was 1.0% (8/782 alleles). In contrast, the c.1393C>T polymorphism was not identified among the individuals who were heterozygous for the c.1001A>G polymorphism or wild-type homozygous.

## Discussion

The patient with severe toxicity after capecitabine treatment was found to be compound heterozygous for polymorphisms c.1001A>G (p.Gln334Arg) and c.1393C>T (p.Arg465Ter). Although we could not determine whether c.1001A>G and c.1393C>T were on different haplotypes in subjects I-6 (proband) and I-2 (proband’s brother), it is likely that these subjects are compound heterozygous from data related to the genotype of subject II-2 (proband’s daughter) (wild-type/c.1393C>T), the genotype of subject II-3 (proband’s son) (wild-type/c.1001A>G), and their urinary dihydrouracil/uracil ratios. The c.1001A>G polymorphism was identified in Japanese DHP-deficient patients and functional analysis has confirmed that mutant DHP enzymes containing the p.Gln334Arg substitution have only 2.5% residual activity [10]. The c.1393C>T mutation was identified in Venezuelan DHP-deficient patients and functional analysis has confirmed that mutant DHP enzymes containing the p.Arg465Ter substitution have no residual activity [23]. Therefore, compound heterozygous mutations c.1001A>G and c.1393C>T should markedly decrease DHP activity.

Sumi et al. analyzed urine samples from 21,200 healthy Japanese infants and found 2 asymptomatic children with dihydropyrimidinuria, which indicated that the incidence of DHP deficiency was 1/10,000 [7]. Hayashi et al. reported that only one man with significant dihydropyrimidinuria was identified in a study of 1,133 Japanese adults [24]. Hamajima et al. found one frameshift and 5 missense polymorphisms of the *DPYS* gene in 6 Japanese patients with dihydropyrimidinuria, and 4 asymptomatic Japanese patients among them were homozygous or heterozygous for the c.1001A>G polymorphism [10]. Our screening analysis using 391 unrelated Japanese subjects indicated that the frequency of the c.1001A>G polymorphism was 1.0%. Another analysis showed that the c.1001A>G polymorphism has an allele frequency of 1.4% in the Japanese population (1000 Genomes; http://www.1000genomes.org/); however, it was not present in other populations. Thus far, 5-FU toxicity has been believed to be caused primarily by DPD deficiency [25], but mass screening results in this study suggest that genetic analysis of the *DPYS* gene is important, at least in Japanese patients.

Severe 5-FU toxicity was reported in single female breast cancer patient with heterozygous missense variant c.833G>A (G278D) in exon 5 of *DPYS* [9], but has not been reported in Japanese patients, or in patients with any homozygous or compound heterozygous polymorphisms who were treated with fluoropyrimidines. In the present study, we provide the first report of a DHP-deficient Japanese patient with severe capecitabine toxicity caused by compound heterozygous polymorphisms c.1001A>G (p.Gln334Arg) in exon 6 and c.1393C>T (p.Arg465Ter) in exon 8. Genetic testing of patients with a pyrimidine degradation defect is important for effective prevention of severe toxicity during treatment with 5-FU or its analogs. It might be possible that 5-FU toxicity appears only in patients with compound heterozygous polymorphisms, as in the current case, and not in cases of single heterozygous polymorphisms, although heterozygous missense variant c.833G>A is thought to be pathogenic in terms of chemotherapy with fluoropyrimidines [9]. If a heterozygous c.1001A>G polymorphism is pathogenic in patients treated with fluoropyrimidines, then urinary dihydrouracil concentration testing cannot be used as a predictive biomarker for toxicity. Further investigation of the clinical relevance of c.1001A>G in the context of fluoropyrimidine toxicity should be conducted in a larger sample. In a recent study, Fidlerova et al. reported that missense and nonsense variants in *DPYS* were infrequent; however, the development of serious gastrointestinal toxicity could be influenced by non-coding *DPYS* sequence variants c.-1T>C and IVS1-58T>C [26]. Further clinical studies, including an analysis of 5-FU and its metabolites in urine and plasma in patients with *DPYS* or *DPYD* polymorphisms such as promoter, intron, and epigenetic variations, will be required to prevent toxicity caused by fluoropyrimidine therapy.

## Supporting Information

S1 Fig Direct sequencing chromatograms of *DPYS* sequence alterations in exon 6 and exon 8 of the patient with severe toxicity after capecitabine treatment. (TIF)

S1 Table PCR primers used to amplify exons of the human *DPYD* gene. (DOCX)

S2 Table PCR primers used to amplify exons of the human *DPYS* gene. (DOCX)
