# Supplementary figures and images for: Genetic Polymorphisms of Dihydropyrimidinase in a Japanese Patient with Capecitabine-Induced Toxicity
Source: PLoS One. 2015 Apr 27;10(4):e0124818. doi: 10.1371/journal.pone.0124818 (PMC4411063; doi:10.1371/journal.pone.0124818)

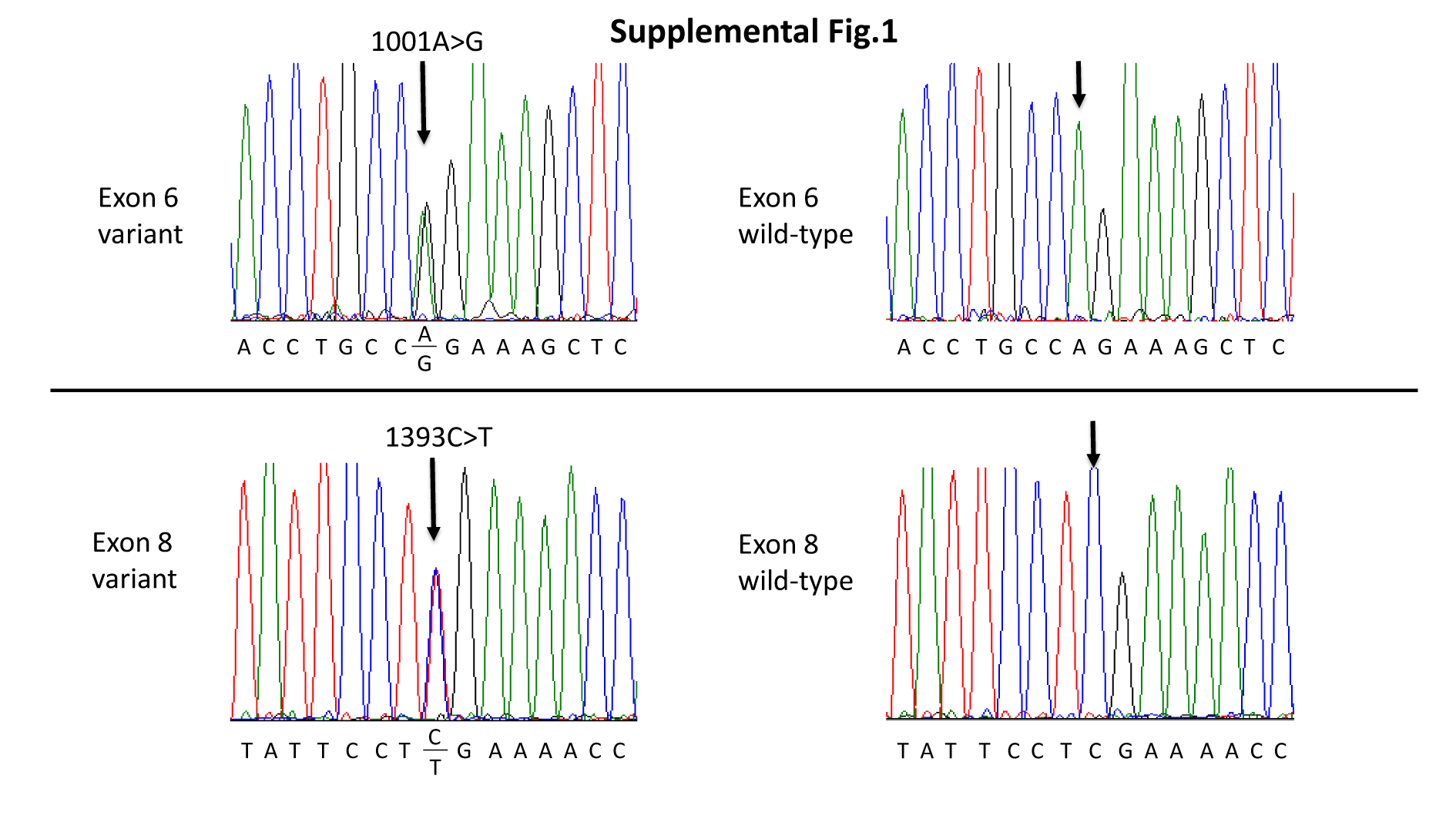

Supplement: S1 Fig — (TIF) [file pone.0124818.s001.tif]
