# Supplementary material for: Genetic Polymorphisms of Dihydropyrimidinase in a Japanese Patient with Capecitabine-Induced Toxicity
Source: PLoS One. 2015 Apr 27;10(4):e0124818. doi: 10.1371/journal.pone.0124818 (PMC4411063; doi:10.1371/journal.pone.0124818)
Supplement: S1 Table — (DOCX) [file pone.0124818.s002.docx]

**Supplemental Table 1.**

PCR primers used to amplify exons of the human *DPYD* gene.

|  | Primer (5′-3′) | | Product length (bp) |
| --- | --- | --- | --- |
| Exon | Sense | Antisense |  |
| 1 | gctgtcacttggctctct | cacctacccgcagagca | 183 |
| 2 | gtgacaaagtgagagagaccgtgtc | gccttacaatgtgtggagtgagg | 285 |
| 3 | gaatgctacccaattaaagtgg | cctaccaccatcctgtgactg | 269 |
| 4 | ggtagaaaatagattatctcact | gaatttaccttgtttgcaatact | 158 |
| 5 | gtttgtcgtaatttggctg | atttgtgcatggtgatgg | 287 |
| 6 | gaggatgtaagctagtttc | ccatttgtgtgcgtgaagttc | 350 |
| 7 | gtcctcatgcatatcttgtgtg | gcttctgcctgatgtagc | 361 |
| 8 | ccttaatagaacatgttcctgt | gcagtcattctggatattgct | 368 |
| 9 | agcccctcctcctgctaat | tgctgctgagcttgattttg | 300 |
| 10 | gatagtgacacttcatcctgg | ctgttggtgtacaactcc | 340 |
| 11 | tggtgaaagaaaaagctgcat | aacagacaattgcatcacaca | 347 |
| 12 | cagttgtttgaatccctggaa | cgcctggcccaatttttaat | 504 |
| 13 | cggatgactgtgttgaagtg | tgtgtaatgataggtcgtgtc | 434 |
| 14 | tgcaaatatgtgaggagggacc | cagcaaagcaactggcagattc | 409 |
| 15 | cccaaatgtcatccagtgt | tttctcatggcagctctttattt | 335 |
| 16 | aacggtgaaagcctattgg | tagtaactatccatacggggg | 223 |
| 17 | cacgtctccagctttgctgttg | cgggcaactgattcaagtcaag | 238 |
| 18 | tgaatgggttttaactatcgtgtc | aagtgggcaacacctaccag | 220 |
| 19 | tgtccagtgacgctgtcatcac | cattgcatttgtgagatggag | 300 |
| 20 | gagaagtgaatttgtttggag | cacagacccatcatatggctg | 399 |
| 21 | cggaacctgataccgagaag | gcagttttcaccatggacag | 476 |
| 22 | gagcttgctaagtaattcagtggc | agagcaatatgtggcacc | 288 |
| 23 | ggggacaatgatgacctatgtgg | ggtgacatgaaagttcacagcaac | 269 |
