# Supplementary material for: Genetic Polymorphisms of Dihydropyrimidinase in a Japanese Patient with Capecitabine-Induced Toxicity
Source: PLoS One. 2015 Apr 27;10(4):e0124818. doi: 10.1371/journal.pone.0124818 (PMC4411063; doi:10.1371/journal.pone.0124818)
Supplement: S2 Table — (DOCX) [file pone.0124818.s003.docx]

**Supplemental Table 2.**

PCR primers used to amplify exons of the human *DPYS* gene.

|  | Primer (5′-3′) | | Product length (bp) |
| --- | --- | --- | --- |
| Exon | Sense | Antisense |  |
| 1 | tgcaggagggcaccccaagc | gaggcggccctgctgaggac | 444 |
| 2 | atgcccttctgggtcattta | tctgtcctcctgttgtctag | 423 |
| 3 | gagcagcagcagtttatcag | gcccaatcatcttcacctta | 454 |
| 4 | gatcaaaagcctggcattgc | ctcctaaactgaagcagagg | 376 |
| 5 | tatctggtagggttttggag | gctcccttctacccaaacct | 532 |
| 6 | gagacaggagagggatgaaa | ggatcctggctgaagaacta | 362 |
| 7 | catcctcagatgctctacaa | ctacatcctctatgccaaga | 476 |
| 8 | tcaagtgagctggtgatgat | ggaaatcccgaactgaccta | 489 |
| 9 | cacaaaaagtggacaatcc | gtgaagcctctgaccttgat | 421 |
